# Advances in molecular biomarkers research and clinical application progress for gastric cancer immunotherapy

**DOI:** 10.1186/s40364-022-00413-0

**Published:** 2022-08-30

**Authors:** Hongzhen Cai, Man Li, Ruiyi Deng, Mopei Wang, Yanyan Shi

**Affiliations:** 1 Research Center of Clinical Epidemiology, Peking University Third Hospital, Beijing, 100191 People’s Republic of China; 2 Peking University Health Science Center, Beijing, 100191 People’s Republic of China; 3 Department of Medical Oncology and Radiation Sickness, Peking University Third Hospital, Beijing, 100191 People’s Republic of China

**Keywords:** Gastric cancer, Immunotherapy, Anti-PD-1, Biomarker, Checkpoint inhibitor

## Abstract

Gastric cancer is characterized by high morbidity and mortality worldwide. Early-stage gastric cancer is mainly treated with surgery, while for advanced gastric cancer, the current treatment options remain insufficient. In the 2022 NCCN Guidelines for Gastric Cancer, immunotherapy is listed as a first-line option for certain conditions. Immunotherapy for gastric cancer mainly targets the PD-1 molecule and achieves therapeutic effects by activating T cells. In addition, therapeutic strategies targeting other molecules, such as CTLA4, LAG3, Tim3, TIGIT, and OX40, have also been developed to improve the treatment efficacy of gastric cancer immunotherapy. This review summarizes the molecular biomarkers of gastric cancer immunotherapy and their clinical trials.

## Background

### Gastric cancer and clinical therapy

Gastric cancer is a condition that significantly affects the quality of life, with high morbidity and mortality worldwide [1]. Currently, gastric cancer is treated using endoscopic therapy, surgical therapy, radiotherapy, chemotherapy, etc. [2]. For early-stage gastric cancer which are usually found on screening (T1a or T1b) treatment, endoscopic therapy can be used [3], and for locoregional gastric cancer, multidisciplinary management is commonly based on surgery [4]. For metastatic and unresectable gastric cancer, radiotherapy, chemotherapy, targeted therapy (HER2/neu [5], VEGFR2 [6], EGFR [7]-based targeted therapy) and immunotherapy are used; however, the prognosis of patients still has much room for improvement [8], as there are still many patients with poor response. Anti-PD-1/PD-L1 immunotherapy has shown potential in the treatment of gastric cancer [9] and has been included in the NCCN 2022 guidelines as a first/second line treatment option [10]. Currently, a number of phase III clinical trials are exploring the efficacy of anti-PD-1/PD-L1 inhibitors in gastric cancer treatment. Other inhibitors or agonists of immune checkpoint molecules, including the inhibitors of CTLA4, LAG3, Tim3, and TIGIT, as well as the agonists of OX40, are currently in clinical trials in the treatment of gastric cancer.

### Characteristics of the gastric mucosal immune microenvironment

During tumor development, the tumor cells rely on blood vessels, fibroblasts, and lymphocytes of the surrounding tissues. These cells and tissues, together with the tumor, are called the tumor microenvironment, in which the immune cells are called the tumor immune microenvironment [11, 12]. In gastric mucosa, inflammatory factors, a variety of lymphocytes, mononuclear macrophages, and gastric epithelial cells participate in the formation of the immune microenvironment [13].

**Fig. 1:**
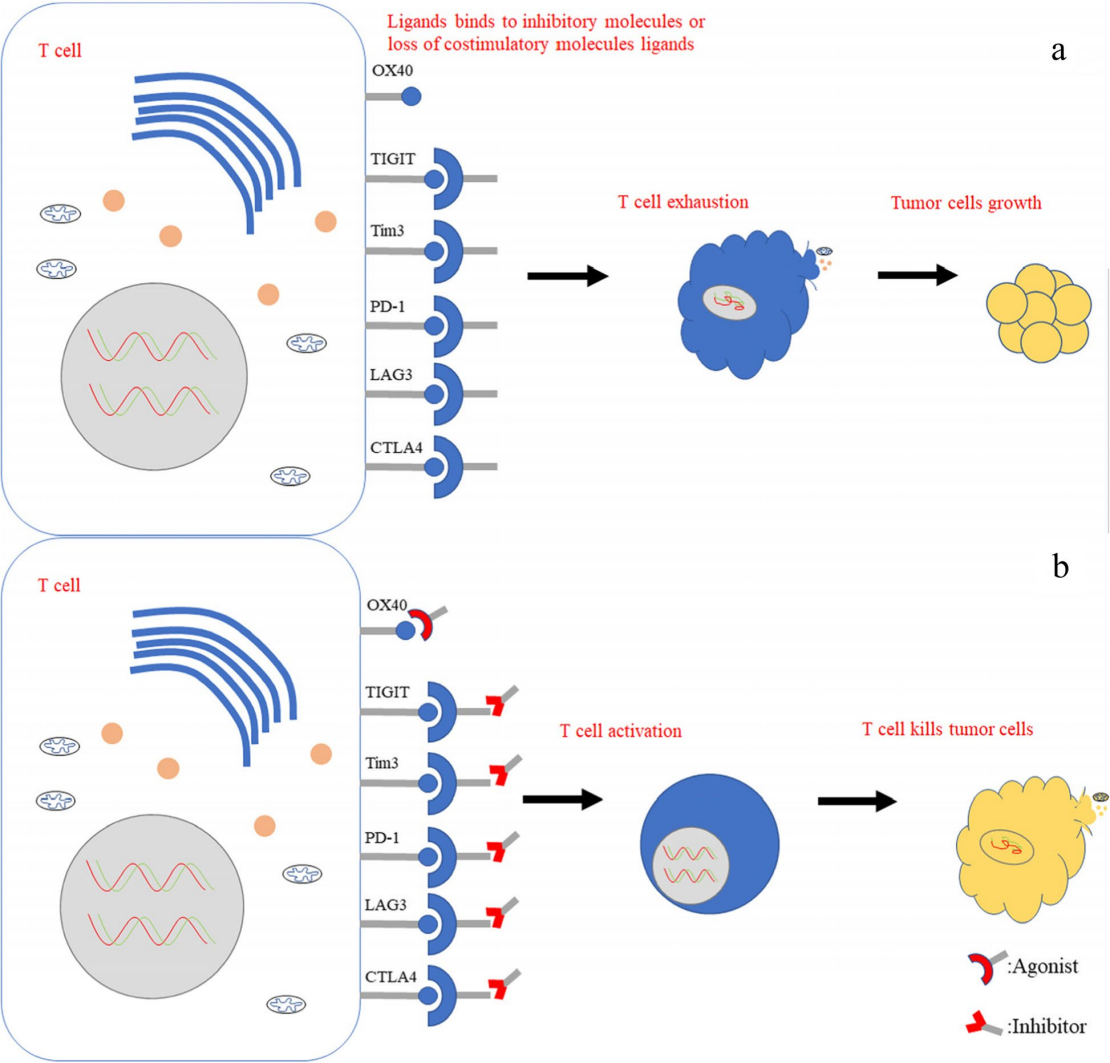
T-cell regulatory biomarkers of immunotherapy for gastric cancer. **a** PD-1, CTLA4, LAG3, Tim3 and TIGIT act as inhibitory molecules, and OX40 acts as a costimulatory molecule. Activation of inhibitory molecules or loss of costimulatory molecules can lead to T-cell exhaustion. **b** The usage of anti-PD-1 inhibitors, etc., or OX40 agonists can activate T cells to kill tumor cells

T lymphocytes play an important role in antitumor immunity, and their functions are regulated by immunosuppressive and costimulatory signals in the tumor microenvironment [14]. Costimulatory molecules of T cells include CD28 and OX40 [15], and immunosuppressive molecules include PD-1, LAG3, Tim3, and TIGIT [16] (Fig. 1).

Programmed death-1 (PD-1), also known as CD279, is an immunosuppressant that is expressed in various immune cells, such as T cells, B cells, and NK cells. The ligand of PD-1 is PD-L1, which is expressed in antigen presenting cells (APC) and tumor cells [17]. The expression level of PD-1 in T cells is influenced by many factors. First, the transcription factors NFATc1, RBP-Jκ, STAT3, STAT4, ISGF3, FoxO1, C-FOS, and NF-κB can promote PD-1 expression, while Blimp-1 and T-bet can inhibit PD-1 expression [18]. Second, posttranscriptional modification can also affect PD-1 levels. For example, ubiquitin modification of PD-1 can degrade it, and glycosylation of PD-1 can affect its binding with anti-PD-1 mAbs [19]. In tumor-infiltrating T lymphocytes, the expression level of PD-1 is high [20], which inhibits the immune surveillance function and leads to the immune escape of tumor cells [21]. Inhibition of PD-L1 in tumor cells and immune cells or PD-1 on T cells can therefore avoid the immune escape of tumor cells [22]. In addition, an animal experiment found that PD-1 knockout on myeloid cells also has antitumor activity [23]. Therefore, anti-PD-1 inhibitors may have potential for use in tumor immunotherapy. In the gastric mucosal immune microenvironment of gastric cancer patients, the proportion of PD-L1 CPS higher than 1 was observed to be 40–60% [24–27]. This may be one of the advantages of immunotherapy for patients with gastric cancer.

Cytotoxic T lymphocyte antigen 4 (CTLA4), also known as CD152, is another immunosuppressive molecule that is expressed on the surface of T cells together with CD28 [28]. The coligand of CTLA4 and CD28 is B7, which is expressed in APCs and tumor cells. When CD28 binds to B7, T cells are activated by this costimulatory signal. When the CTLA4 pathway binds to B7, T cells receive inhibitory signals and are exhausted [29]. With higher affinity for B7 than CD28, excessive activation of the CTLA4 pathway leads to a decline in immune function and subsequent immune escape of tumor cells [30]. Hence, anti-CTLA4 inhibitors can potentially be used to activate T cells and kill tumor cells. Ipilimumab, as an anti-CTLA4 antibody, when combined with nivolumab, has been shown to be highly effective for dMMR gastric cancer (ORR 70, 95% confidence interval 35, 93 versus 57, 95% confidence interval 18, 90) [31]. This result shows that ipilimumab can be a promising agent for gastric cancer immunotherapy.

Lymphocyte activation gene-3 (LAG3), also known as CD233, is an immunosuppressive molecule that belongs to the immunoglobulin superfamily and inhibits T cells. LAG3 was first discovered on the surface of activated T cells and NK cells by Triebel et al. in 1990 [32]. In addition, LAG3 has also been found to be expressed on the surface of B cells, tumor-associated macrophages, and other cells. LAG3 includes soluble sLAG3 and membrane mLAG3, in which the D1 domain of sLAG3 binds MHC class II molecules [33]. LAG3 binding MHC class II molecules can transmit inhibitory signals through its intracellular domain [34], but the molecular mechanisms of this effect are not yet clear. In addition to MHC class II molecules, other ligands of LAG3 have been found, such as Gal9, LSECtin, and FGL1, all of which play certain functions in T-cell exhaustion [35]. As the activation of LAG3 has an inhibitory effect on T cells, the use of anti-LAG3 inhibitors to activate the immune system and kill tumor cells represents another potential immunotherapeutic strategy.

T-cell immunoglobulin and mucin domain-3 (Tim3), another immunosuppressive molecule, is expressed on the surface of T cells, myeloid cells, dendritic cells, mast cells and NK cells [36]. Tim3 can bind to phosphatidylserine (PS) and inhibit the activation of NK cells. The use of photosensitive PS has been shown to regulate the activity of NK cells in vivo and in vitro [37]. In addition, Tim3 plays an auxiliary diagnostic or monitoring role in HIV infection [38] and tuberculosis infection [39], suggesting that it is associated with the immune regulation of the body. The ligand of Tim3 is Gal9, and studies have shown that the Tim3-Gal9 pathway can inhibit Th1-cell activation [40]. Moreover, Tim3 is induced in regulatory T cells (Tregs) and inhibits the function of effector T cells [41]. These studies reveal the inhibition of Tim3, which negatively regulates T cells, as another potential immunotherapeutic strategy.

The immunosuppressive T-cell immunoglobulin and ITAM domain (TIGIT) is expressed on the surface of T cells and NK cells. The ligand of TIGIT is CD155, which is the coligand of TIGIT and CD226. While the CD226 molecule binds to CD155 to activate immune cells, TIGIT binds to CD155 more strongly and competes with CD226, leading to a negative effect. The binding of CD155 by TIGIT inhibits immune cells through the PI3K, MAPK, and NF-κB signaling pathways [42]. A study has shown that blockade of TIGIT in mice can enhance antitumor immunity in an NK-dependent manner [43], suggesting the therapeutic value of anti-TIGIT inhibitors in cancer.

Siglec-15 is also a newly discovered membrane protein that is expressed on the surface of macrophages/myeloid cells and cancer cells and can inhibit T-cell responses. Studies have found that a Siglec-15 inhibitor can reverse this effect and enhance T-cell immunity, leading to tumor suppression in a mouse model [44]. This finding suggests that Siglec-15 may serve as a novel immunotherapy biomarker. No clinical trials are designed for gastric cancer immunotherapy, and further studies are needed.

OX40 is a T-cell costimulatory molecule and a member of the tumor necrosis factor receptor superfamily, also known as CD134. OX40 can be expressed on the surface of T cells. Activation of the OX40/OX40L pathway can reverse T-cell depletion, increase the proportion of CD4/CD8-positive T cells, and enhance the immune response of the body. In addition to activating T cells, OX40 is also found in normal glandular tissue cells and tumor cells [45]. However, the role of OX40 expression in these cells still needs further study. OX40 is associated with autoimmune diseases such as psoriasis, which has been treated with the anti-OX40 mAb in clinical studies [46, 47]. Considering that abatacept (exogenous CTLA4) is a drug for the treatment of autoimmune diseases, ipilimumab (anti-CTLA4) is an antitumor drug, and OX40 inhibitor is used for treating autoimmune diseases, it is speculated that the OX40 agonist receptor may also have antitumor activity. Clinical studies have found that the expression level of OX40 in the gastric mucosal immune microenvironment of gastric cancer patients is increased, and the OX40 expression level of stage I and II gastric cancer patients is higher than that of stage III and IV gastric cancer patients [48], suggesting that the immune system of patients with advanced gastric cancer is suppressed and that OX40 may play an important role in the immune microenvironment of gastric cancer.

*H. pylori* is a gram-negative bacterium found on the surface of gastric epithelial cells [49]. In many parts of the world, the prevalence of *H. pylori* is higher than 50% [50]. *H. pylori* has been identified as a class I carcinogen of gastric cancer by the WHO, promoting gastric cancer through virulence factors, such as CagA, TNF-α-induced protein, and YAP-mediated epithelial metaplasia (EMT) [51]. *H. pylori* infection can affect the gastric mucosal immune microenvironment and induce the expression of PD-L1 in gastric epithelial cells, enabling these cells to survive chronic inflammation and giving them the potential to become cancerous [52]. In the gastric mucosa of gastric cancer patients, the expression level of PD-1/PD-L1 is significantly higher than in the general population [53], and in the gastric mucosa of early-stage gastric cancer patients and high-grade gastric intraepithelial neoplasia infected with *H. pylori*, the expression level of PD-L1 is also significantly higher than that of patients with low-grade gastric intraepithelial neoplasia [54]. These results suggest that *H. pylori* may be an important factor in the immunotherapy of gastric cancer, and further research is still needed, as it remains unclear whether the immunotherapy biomarker of gastric cancer interacts with *H. pylori*.

## Clinical research status of gastric cancer immunotherapy biomarkers

Studies on biomarkers for immunotherapy of gastric cancer have mainly focused on PD-1/PD-L1, while there are also many other biomarkers that have potential for use in the immunotherapy of gastric cancer (Table 1).

**Table 1:** Clinical research progress of immunotherapy for gastric cancer

Biomarker	Inhibitor	Study	Clinical trials	Phase	Therapeutic strategy	Group	Treatment effect
PD-1	Nivolumab	Kang YK et al. [52]	**ATTRACTION-4**	Phase 3	first-line	nivolumab plus chemotherapy vs placebo plus chemotherapy	ORR: 57.5% vs 47.8%
		Janjigian YY et al. [53]	**CheckMate 649**	Phase 3	first-line	nivolumab plus chemotherapy vs chemotherapy	ORR: 60% vs 45% in PD-L1 CPS > =5
		Kang YK, Boku N et al. [54, 55]	**ATTRACTION-2**	Phase 3	third-line	nivolumab vs placebo	ORR: 32% vs 0%
		Kelly RJ et al. [56]	**CheckMate 577**	Phase 3	adjuvant	nivolumab vs placebo	ORR not tested PFS: 22.4 vs 11.0 months
		Fukuoka S et al. [57]	**EPOC1603**	Phase 1	second/third-line	nivolumab plus regorafenib	ORR: 44% in gastric cancer
	Pembrolizumab	Shitara K et al. [58]	**KEYNOTE-062**	Phase 3	first-line	pembrolizumab vs pembrolizumab plus chemotherapy vs chemotherapy	ORR: 15% vs 49% vs 37%
		Janjigian YY et al. [59]	**KEYNOTE-811**	Phase 3	first-line	pembrolizumab plus trastuzumab plus chemotherapy vs placebo plus trastuzumab plus chemotherapy	ORR: 74.4% vs 51.9%
		Shitara K,Fuchs CS et al. [60, 61]	**KEYNOTE-061**	Phase 3	second-line	pembrolizumab vs paclitaxel	ORR: 17.3% vs 15.6% in PD-L1 CPS > =1
		Chung HC et al. [62]	**KEYNOTE-063**	Phase 3	second-line	pembrolizumab vs paclitaxel	ORR: 13% vs 19%
		Marabelle A et al. [63]	**KEYNOTE-158**	Phase 2	second/third-line	pembrolizumab	ORR: 34.3%
		Kawazoe A et al. [64]	**EPOC1706**	Phase 2	first/second-line	pembrolizumab plus lenvatinib	ORR: 69%
	Dostarlimab-gxly	https://clinicaltrials.gov/ct2/show/NCT02715284	**GARNET**	Phase 1	second-line	dostarlimab	Ongoing
Biomarker	Inhibitor	Study	Clinical trials	Phase	Therapeutic strategy	Group	Treatment effect
CTLA4	Ipilimumab	Shitara K et al. [65]	**CheckMate 649**	Phase 3	first-line	nivolumab plus ipilimumab vs chemotherapy	ORR: 70% vs 57% in MSI-H tumors
		Janjigian YY et al. [66]	**CheckMate 032**	Phase 1/2	third-line	nivolumab 3 mg/kg vs nivolumab 1 mg/kg plus ipilimumab 3 mg/kg vs nivolumab 3 mg/kg plus ipilimumab 1 mg/kg	ORR: 12% vs 24% vs 8%
		Bang YJ et al. [67]	**NCT01585987**	Phase 2	second-line	nivolumab vs best supportive care	ORR: 1.8% vs 7%
		Tintelnot J et al. [68]	**INTEGA**	Phase 2	first-line	ipilimumab plus nivolumab plus trastuzumab vs chemotherapy plus nivolumab plus trastuzumab	Ongoing
	Tremelimumab	Ralph C et al. [69]	researcher **Christy Ralph**	Phase 2	second-line	tremelimumab	ORR: 5%
		Raimondi A et al. [70]	**INFINITY**	Phase 2	neoadjuvant	tremelimumab and durvalumab	Ongoing
		Evrard C et al. [71]	**DURIGAST**	Phase 2	second-line	tremelimumab plus durvalumab plus chemotherapy vs durvalumab plus chemotherapy	
LAG3	MbS-986,213	https://clinicaltrials.gov/ct2/show/NCT03662659	**NCT03662659**	Phase 2	first-line	MbS-986,213 plus chemotherapy	Ongoing
	Relatlimab	https://clinicaltrials.gov/ct2/show/NCT03044613	**NCT03044613**	Phase 1	adjuvant	relatlimab plus nivolumab plus chemotherapy	
	Tebotelimab	https://clinicaltrials.gov/ct2/show/NCT04082364	**NCT04082364**	Phase 2/3	first-line	margetuximab plus tebotelimab plus chemotherapy	
Tim3	INCAGN02390	https://clinicaltrials.gov/ct2/show/NCT03652077	**NCT03652077**	Phase 1	first-line	INCAGN02390	Ongoing
TIGIT	Tiragolumab	https://clinicaltrials.gov/ct2/show/NCT04933227	**NCT04933227**	Phase 2	first-line	tiragolumab plus atezolizumab plus chemotherapy	Ongoing
		https://clinicaltrials.gov/ct2/show/NCT05251948	**NCT05251948**	Phase 2	second-line	tiragolumab plus atezolizumab plus chemotherapy	Recruiting
OX40	INBRX-106	https://clinicaltrials.gov/ct2/show/NCT04198766	**NCT04198766**	Phase 1	second/third-line	INBRX-106 with or without pembrolizumab	Recruiting

### PD-1

Nivolumab, pembrolizumab and dostarlimab-gxly have been included in the 2022 NCCN guidelines for the treatment of gastric cancer.

Nivolumab (anti-PD-1) is currently a first-line agent in the guidelines for the treatment of gastric cancer. ATTRACTION-4 and CheckMate 649 studied the efficacy of nivolumab as a first-line agent. In the multicenter, double-blind, placebo-controlled, randomized controlled phase II/III trial ATTRACTION-4 involving 724 patients with HER2-negative, unresectable, advanced or relapsed gastric cancer or gastroesophageal junction cancer treated with nivolumab in combination with chemotherapy (*n* = 362) or placebo in combination with chemotherapy (*n* = 362), the median PFS was 10.45 months in the nivolumab group and 8.34 months in the placebo group (*P* = 0.0007) at the 11.6-month median follow-up. At the 26.6-month median follow-up, the median OS was 17.45 months in the nivolumab group and 17.15 months in the placebo group (*P* = 0.26) [55]. Another multicenter, open-label, randomized controlled phase III clinical trial, CheckMate 649, enrolled 1581 patients with unresectable, non-HER2-positive gastric or gastroesophageal junction cancer or esophageal adenocarcinoma for first-line therapy. Patients were treated with nivolumab in combination with chemotherapy (*n* = 789) or chemotherapy alone (*n* = 792). The median follow-up OS was 13.1 months in the nivolumab group and 11.1 months in the chemotherapy group. In patients with PD-L1 CPS ≥ 5, both OS (*P* < 0.0001) and PFS (*P* < 0.0001) in the nivolumab group were better than those in the chemotherapy group [56]. For third-line treatment, a double-blind, placebo-controlled, randomized controlled phase III trial ATTRACTION-2 recruited 493 patients with unresectable metastatic or relapsed gastric cancer or gastroesophageal junction cancer who were treated with either nivolumab (*n* = 330) or placebo (*n* = 163), and the three-year follow-up data showed a median OS of 5.26 months in the nivolumab group versus 4.14 months in the placebo group (*P* < 0.0001) [57, 58]. For adjuvant treatment, the CheckMate 577 double-blind, placebo-controlled, randomized controlled phase III clinical trial treated patients with esophageal cancer or gastroesophageal junction cancer after R0 resection with nivolumab (*n* = 532) or placebo (*n* = 262), and the median PFS was 22.4 months in the nivolumab group and 11.0 months in the placebo group (*P* < 0.001) [59]. The open-label phase I trial EPOC1603 performed on 50 patients with advanced gastric cancer (*n* = 25) or colorectal cancer (*n* = 25) treated with nivolumab combined with regorafenib (chemotherapy agent, tyrosine kinase inhibitor) for second/third-line therapy demonstrated its safety [60].

Pembrolizumab (anti-PD-1) is currently a first/second-line agent in the guidelines for the treatment of gastric cancer. For HER2 overexpression-positive adenocarcinoma, chemotherapy plus trastuzumab plus pembrolizumab is a first-line therapy. For MSI-H or dMMR tumors and for TMB high (≥10 mutations/megabase) tumors, pembrolizumab can be used as a second-line agent. KEYNOTE-062 and KEYNOTE-811 studied the efficacy of pembrolizumab as a first-line agent. In the randomized controlled phase III trial KEYNOTE-062, 763 patients with advanced gastric or gastroesophageal junction cancer with PD-L1 CPS ≥ 1 were treated with pembrolizumab (*n* = 256), pembrolizumab in combination with chemotherapy (*n* = 257) or chemotherapy in combination with placebo (*n* = 250). At a median follow-up time of 29.4 months, it was observed that pembrolizumab had a better effect than chemotherapy in prolonging OS in patients with CPS ≥ 10 (17.4 vs 10.8 months), but no significant difference was observed between the median OS time of other groups [61]. In a randomized, double-blind, placebo-controlled phase III trial, KEYNOTE-811 recruited 264 patients with previously untreated unresectable or metastatic, HER2-positive gastric or gastroesophageal junction adenocarcinoma for efficacy evaluation, 133 of whom received pembrolizumab in combination with trastuzumab and chemotherapy, while 131 of whom received placebo in combination with trastuzumab and chemotherapy. It was observed that the ORRs were 74.4 and 51.9% in the pembrolizumab group and placebo group, respectively (*p* = 0.00006) [62]. KEYNOTE-061 and KEYNOTE-063 studied the efficacy of pembrolizumab as a second-line agent. The open-label, randomized controlled phase III trial KEYNOTE-061 treated 395 advanced gastric or gastroesophageal junction cancer patients with a PD-L1 CPS ≥ 1 with pembrolizumab (*n* = 196) or paclitaxel (*n* = 199). The median OS time was observed to be 9.1 months in the pembrolizumab group versus 8.3 months in the paclitaxel group (*P* = 0.0421) [63]. In addition, the two-year follow-up results of KEYNOTE-061 showed no significant difference in the effect of pembrolizumab and paclitaxel on OS. However, the difference in the 24-month OS rate between the two groups increased with increasing PD-L1 CPS. In the CPS ≥ 5 group, the 24-month OS rate was 24.2% for pembrolizumab and 8.8% for paclitaxel. In the CPS ≥ 10 group, the 24-month OS rate was 32.1% for pembrolizumab and 10.9% for paclitaxel, suggesting that patients with high CPS were more likely to have a better response to pembrolizumab [64]. An open-label, randomized, controlled phase III clinical trial, KEYNOTE-063, was performed on 94 patients with advanced gastric cancer or gastroesophageal junction cancer treated with pembrolizumab (*n* = 47) or paclitaxel (*n* = 47), and the effect of pembrolizumab was observed to be weaker than that of paclitaxel [72]. In the open-label, nonrandomized controlled phase II clinical trial KEYNOTE-158, an ORR of 34.3% was observed in 233 patients with advanced or unresectable noncolorectal MSI-H cancer treated with pembrolizumab for second/third-line therapy [73]. In addition, an open-label, single-arm phase II trial EPOC1706 involving 29 patients with metastatic or recurrent gastric or gastroesophageal junction cancer demonstrated the safety of pembrolizumab when used in combination with lenvatinib (chemotherapy, tyrosine kinase inhibitor) as first/second-line treatment and found an ORR of 69% [74]. In conclusion, pembrolizumab in gastric cancer has a promising therapeutic effect as a first-line agent, but its effectiveness needs to be further studied when used as a second-line agent.

Dostarlimab-gxly was first included in the 2022 NCCN guidelines for gastric cancer as a second-line treatment agent in certain circumstances for MSI-H or dMMR tumors. However, the clinical trials for gastric cancer immunotherapy only progressed to phase I/II. The multicenter, open-label, single-arm phase 1 trial GARNET recruited 740 patients with advanced solid tumors who have limited available treatment options to date (https://clinicaltrials.gov/). Given that 42.3% of patients with MSI-H endometrial cancer receiving dostarlimab had an objective response, dostarlimab may be an alternative for MSI-H solid tumors [75].

The efficacy of anti-PD-1/PD-L1 immunotherapy is dependent on many factors, and there is still room for improvement. First, studies found that the expression of PD-L1 is an important factor influencing the efficacy of gastric cancer immunotherapy. In Checkmate 649, patients with PD-L1 CPS ≥ 10, ≥5 and ≥ 1 received nivolumab plus chemotherapy, resulting in HRs of 0.66, 0.69 and 0.74, respectively [56]. The results of KEYNOTE-061 suggested that the CPS level of PD-L1 was correlated with the clinical efficacy of gastric cancer immunotherapy, and a favorable effect on OS was observed in the subgroups of CPS ≥ 1 and CPS ≥ 10 [76]. In KEYNOTE-659, the ORR was 73.9% for patients with 1 ≤ CPS ≤ 10 and 71.0% for patients with CPS ≥ 10 [77]. In the NCT02589496 study of 55 patients with gastric cancer whose PD-L1 CPS positivity was available (61 gastric cancer patients in total), 28 PD-L1 CPS ≥ 1 patients had an ORR of 50%, while 27 PD-L1 CPS ≤ 1 patients had an ORR of 0% [78]. In the 2022 NCCN guidelines for the treatment of gastric cancer, PD-L1 testing may be considered for gastric cancer patients with anti-PD-1 immunotherapy treatment. Therefore, attention should be given to detecting the expression level of PD-L1 before using anti-PD-1/PD-L1 inhibitors in the treatment of gastric cancer.

Second, *H. pylori* has been found to cause gastric cancer because it affects the gastric mucosal immune microenvironment of gastric cancer patients. There have been no clinical trials comparing the relationship between *H. pylori* infection and the efficacy of immunotherapy for gastric cancer; however, a study involving melanoma and colorectal adenocarcinoma mice and non-small cell lung cancer patients found that *H. pylori* reduced the efficacy of anti-PD-1 immunotherapy [79]. In addition, an animal experiment found that a recombinant plasmid formed by the *H. pylori* virulence genes CagA, VacA, and BabA could induce CD3+ T cells in animals and had antitumor activity in both in vivo and in vitro experiments [65], suggesting that *H. pylori* infection may be an important factor influencing the efficacy of gastric cancer immunotherapy.

In addition, microsatellite instability (MSI) levels have also been associated with the efficacy of anti-PD-1/PD-L1 immunotherapy. In the case of mismatch repair gene defects (dMMR), the MSI is high (MSI-H), whereas with proficiency mismatch repair gene (pMMR), the condition is described as MSI-Low (MSI-L) or microsatellite-stable (MSS). Gastric cancer patients with MSI-H are highly responsive to immunotherapy. In KEYNOTE-059, the response rate of MSI-H patients to PD-1/PD-L1 immunotherapy was 57% (13.3% in the control group). In the NCT02589496 cohort A study involving 61 gastric cancer patients, the response rate to pembrolizumab of seven MSI-H gastric cancer patients was 85.7% [78]. In the NCT02589496 cohort B study, the response rate to pembrolizumab of eighteen MSI-H gastric cancer patients was 55.6% [66]. Therefore, MSI-H may have a favorable effect on the immunotherapy of gastric cancer.

Moreover, in a study of 61 patients with gastric cancer in NCT02589496, the ORR of six EBV-positive patients was observed to be 100% [78], suggesting that EBV infection is also a favorable factor for the efficacy of gastric cancer immunotherapy.

A study of 36 patients with gastric cancer found that approximately 10% of patients treated with anti-PD-1 inhibitors experienced a more than twofold increase in tumor growth rate and a more than 50% increase in tumor load. This study also found that in animal experiments, anti-PD-1 inhibitors can promote the proliferation of Tregs and inhibit the body’s immunity, thus resulting in the phenomenon called “hyperprogression”. This process can be inhibited by anti-CLTA4 inhibitors [67]. These results suggest that anti-PD-1 inhibitors combined with other immunotherapeutic drugs may provide benefits to patients.

### CTLA4

Anti-CTLA4 immunotherapy has not yet entered the 2022 guidelines for gastric cancer, but the effect of ipilimumab and tremelimumab has been studied in clinical trials.

Clinical trials of ipilimumab in the immunotherapy of gastric cancer have progressed to phase III. Subsequent to CheckMate 649, Kohei Shitara et al. recruited 813 patients with unresectable advanced or metastatic gastric, GEJ or esophageal adenocarcinoma, 409 of whom were treated with nivolumab plus ipilimumab and 404 of whom were treated with chemotherapy. The median OS was 11.2 months versus 11.6 months, respectively, in PD-L1 CPS ≥ 5 patients and 11.7 months versus 11.8 months, respectively, in all randomized patients, showing no statistical significance. However, in patients with MSI-H tumors, nivolumab plus ipilimumab had a high ORR compared with chemotherapy (70% versus 57%) [31]. In CheckMate 032, 160 patients with locally advanced or metastatic gastric or gastroesophageal junction cancer were treated with different doses of ipilimumab combined with nivolumab for third-line therapy (1. nivolumab 3 mg/kg, *n* = 59; 2. nivolumab 1 mg/kg combined with ipilimumab 3 mg/kg, *n* = 49; 3. nivolumab 3 mg/kg combined with ipilimumab 1 mg/kg, *n* = 42), and the OS proportions at 12 months were 39, 35 and 24%, respectively. The proportions of PFS were 8, 17 and 10%, respectively [68]. In the open-label phase II clinical trial NCT01585987, 114 patients with locally advanced or metastatic gastric or gastroesophageal junction cancer were randomly divided into two groups, with 57 patients in each group. The immune-related PFS was 2.92 for ipilimumab and 4.9 for optimal supportive treatment (*P* = 0.097), showing no difference between the two groups when used as second-line therapy [69]. In addition, an open-label, multicenter phase II clinical trial INTEGA involving first-line ipilimumab in combination with nivolumab and trastuzumab is ongoing [70].

Clinical trials of tremelimumab in the immunotherapy of gastric cancer have advanced to phase II. A phase II clinical trial (researcher Christy Ralph) of 18 patients with metastatic gastroesophageal carcinoma showed that after second-line tremelimumab treatment, the median survival time was 17.1 months in patients with a carcinoembryonic antigen proliferative response and 4.7 months in patients without a response (*P* = 0.004) [71]. In addition, multicenter phase II clinical trials INFINITY for neoadjuvant tremelimumab plus durvalumab (anti-PD-L1) and DURIGAST for second-line tremelimumab in combination with durvalumab and chemotherapy are ongoing [80, 81].

It is worth noting that both anti-PD-1 inhibitors and anti-CTLA4 inhibitors have certain side effects in clinical application, which may involve the cardiovascular, endocrine, digestive, blood, and nervous systems and may cause systemic, autoimmune, or skin diseases [82]. A recent animal study showed that avoiding the lysosomal degradation of CTLA4 attenuates the occurrence of immunotherapy-related adverse reactions (irAEs) [83]. The control of side effects is an important task in tumor immunotherapy and needs further study.

### LAG3

Studies on anti-LAG3 inhibitors in gastric cancer are currently in phase I/II clinical trials, without any published results. One of these studies is the open-label, randomized, controlled phase II trial NCT03662659 using MbS-986,213 (relatlimab together with nivolumab) in combination with chemotherapy in comparison to nivolumab alone in combination with chemotherapy for first-line therapy in 274 patients with locally advanced or metastatic gastroesophageal cancer. Another ongoing study is the nonrandomized controlled phase I trial NCT03044613 enrolling 32 patients with gastroesophageal cancer who were treated with relatlimab in combination with nivolumab and chemotherapy in comparison to nivolumab in combination with chemotherapy for adjuvant therapy. In addition, the open-label, randomized controlled phase II/III clinical trial NCT04082364 using tebotelimab (anti PD-1 and anti-LAG3) as first-line therapy is ongoing (https://clinicaltrials.gov/).

Although LAG3 inhibitors are still in phase I/II clinical trials regarding the immunotherapy of gastric cancer and the efficacy is uncertain, clinical trial results of melanoma provide some about the potential effect of anti-LAG3 inhibitors in gastric cancer immunotherapy. In the double-blind, randomized controlled phase II/III clinical trial RELATIVITY-047, the PFS time of the anti-LAG3 inhibitor relatlimab combined with nivolumab was 10.1 months, compared to 4.6 months in the nivolumab group (*P* = 0.006) [84], demonstrating the value of anti-LAG3 inhibitors in immunotherapy.

In addition, clinical studies have found that among patients receiving gastric cancer immunotherapy (nivolumab ± ipilimumab [85], nivolumab [86]), patients with high expression of LAG3 in the gastric mucosal immune microenvironment have a better prognosis. This may be associated with the positive correlation between the expression of LAG3 on the surface of T cells and the expression level of PD-1/PD-L1 in the immune microenvironment of esophageal cancer [87], gastric cancer [88], and other tumors. A cell experiment also demonstrated the strong cytotoxicity of anti-LAG3 inhibitors as single drugs or combined with anti-PD-1/PD-L1 immunotherapy on gastric cancer cells [89]. These results reveal the potential value of anti-LAG3 inhibitors in gastric cancer immunotherapy.

Previous studies have analyzed the relationship between the LAG3 expression level and the diagnosis and prognosis of gastric cancer. First, the expression level of LAG3 in peripheral blood was shown to be closely associated with TNM stage, depth of invasion, and degree of histological differentiation of gastric cancer, revealing the LAG3 expression level as a promising biomarker for the diagnosis of gastric cancer [90]. In addition, high expression of LAG3 in the gastric mucosal immune microenvironment suggests poor prognosis of gastric cancer [91]. High expression of LAG3 was found in patients who did not respond to chemotherapy [92]. Increased LAG3 expression was also observed in patients undergoing gastric cancer surgery [93, 94].

Therefore, LAG3, a newly discovered immunosuppressive molecule, may be an important factor influencing the efficacy of gastric cancer immunotherapy, and anti-LAG3 inhibitors may have potential for developing novel immunotherapeutic strategies for the treatment of gastric cancer.

### Tim3

Anti-Tim3 inhibitors are currently in phase I clinical trials regarding the treatment of gastric cancer. The open-label phase I trial NCT03652077 enrolled 40 patients with advanced cancer using INCAGN02390 (an anti-Tim3 inhibitor) as first-line therapy to study its safety, tolerability, and initial efficacy (https://clinicaltrials.gov/).

In the immune microenvironment of gastric cancer tissues, the expression level of Tim3 is significantly higher than that in normal gastric mucosa tissues [95, 96], and the expression level of Tim3 was shown to be positively correlated with PD-1/PD-L1 [88], suggesting that Tim3 may have an effect on anti-PD-1/PD-L1 immunotherapy.

Currently, few clinical studies have been conducted on the use of anti-TIM3 inhibitors in the treatment of gastric cancer. The potential benefits of anti-Tim3 inhibitors for gastric cancer patients and their potential for use in combination with anti-PD-1/PD-L1 inhibitors in the treatment of gastric cancer need further research.

### TIGIT

The study of anti-TIGIT inhibitors in gastric cancer treatment is in its infancy. The open-label phase II trial NCT04933227 is scheduled to treat 60 patients with metastatic or relapsed gastric or gastroesophageal junction cancer with atezolizumab (anti-PD-L1) and tiragolumab (anti-TIGIT) in combination with chemotherapy as first-line therapy. The open-label, multicenter, randomized controlled phase I/II trial NCT05251948 is scheduled to treat 90 patients with advanced gastric or gastroesophageal junction cancer with atezolizumab (anti-PD-L1) in combination with chemotherapy and tiragolumab (anti-TIGIT) as second-line therapy. The primary end point of both studies was the ORR (https://clinicaltrials.gov/).

### OX40

OX40 agonists are also in the early stages of research in gastric cancer treatment. An open-label, nonrandomized, controlled phase I trial, NCT04198766, is currently in the recruitment phase to study the safety and tolerable dosage of the OX40 agonist INBRX-106-Hexavalent, alone or in combination with pembrolizumab, in 200 patients with advanced solid tumors, including gastric cancer, as second/third-line therapy.

## Discussion

Immunotherapy is an important treatment option for gastric cancer and has been widely studied. Currently, the immunotherapy drugs nivolumab and pembrolizumab are included in the treatment guidelines for gastric cancer, both of which are PD-1 inhibitors that activate the immune system by downregulating the function of immunosuppressive molecules, resulting in enhanced killing of tumor cells. The effectiveness of anti-PD-1/PD-L1 inhibitors in gastric cancer immunotherapy is dependent on a variety of factors, among which a high PD-L1 CPS may be a factor favorably influencing the efficacy of gastric cancer immunotherapy. Although a high PD-L1 CPS (with a cutoff CPS value of 1) has shown a significant difference in prolonging OS, studies have reported that anti-PD-1/PD-L1 inhibitor treatment has shown consistent long-term clinical efficacy in a variety of tumors regardless of PD-L1 expression [97]. Other risk factors, including MSI-H and EBV infection, may also be associated with increased gastric cancer immunotherapy effectiveness, whereas the effect of *H. pylori* infection needs to be further determined. Current immunotherapeutic options do not cover all populations, and our knowledge of factors associated with better outcomes remains inadequate. Therefore, further studies aimed at unraveling the associated molecular pathways and finding new influential factors for immunotherapy are vital.

Based on existing research, we can predict the factors favorable for successful immunotherapy and thereby determine the population suitable for immunotherapy. For patients who did not benefit from immunotherapy, we can explore the potential factors associated with the low effectiveness based on the biological characteristics of the tumor, including different kinds of gene mutations and abnormal activation of signal pathways, as well as using information regarding the patient’s tumor microenvironment, the general condition of the patient, and the lifestyle of the patient [98].

To effectively study biomarkers, new methods are also needed. Omics involves the systematic collection of data from certain groups of individuals and has been widely used in many areas. Whether metabolomics, gut microbiome analysis, and other novel methods can also be used to study the biological factors affecting tumors needs to be investigated [99, 100]. Using mathematical models is a novel way to try to understand the mechanism of cancer development, growth and prognosis and may also have potential for use in cancer immunotherapy [101].

Immunosuppressive molecules such as CTLA4, LAG3, Tim3, and TIGIT may have the same or similar effects as PD-1. Clinical studies involving inhibitors of these molecules have progressed to phase I/II clinical trials. These trials cover the single-agent safety and antitumor efficacy of immunosuppressive molecular inhibitors, as well as their efficacy in combination with chemotherapeutic agents or anti-PD-1 inhibitors. In addition, the expression levels of LAG3 and Tim3 in gastric mucosa may be associated with the efficacy of anti-PD-1 inhibitor immunotherapy. Novel molecular biomarkers, such as Siglec-15, bring new possibilities to cancer immunotherapy. The discoveries and research (including animal experiments and clinical trials) regarding such novel molecules should be boosted. The results of these studies will provide new clues for the use of anti-PD-1 inhibitors and immunotherapy for gastric cancer, which may bring more benefits to patients and improve their prognoses. Regarding the costimulatory molecule OX40, its agonist has entered phase I clinical trials in gastric cancer treatment, from which we expect to see more beneficial results for patients.

Future studies on immune checkpoint inhibitors are required to understand the mechanisms through which these factors influence the efficacy of immunotherapy and to enhance the responses that are beneficial for gastric cancer patients.

## Data Availability

The datasets used and/or analyzed during the current study are available from the corresponding author on reasonable request.
